# Hypoxic Respiratory Chemoreflex Control in Young Trained Swimmers

**DOI:** 10.3389/fphys.2021.632603

**Published:** 2021-02-26

**Authors:** Alexis Arce-Álvarez, Carlos Veliz, Manuel Vazquez-Muñoz, Magdalena von Igel, Cristian Alvares, Rodrigo Ramirez-Campillo, Mikel Izquierdo, Gregoire P. Millet, Rodrigo Del Rio, David C. Andrade

**Affiliations:** ^1^Escuela de Kinesiología, Facultad de Salud, Universidad Católica Silva Henríquez, Santiago, Chile; ^2^Centro de Investigación en Fisiología del Ejercicio, Facultad de Ciencias, Universidad Mayor, Santiago, Chile; ^3^Navarrabiomed, Complejo Hospitalario de Navarra (CHN), Universidad Pública de Navarra, IdiSNA, Pamplona, Spain; ^4^Unidad de Estadística, Departamento de Calidad, Clínica Santa María, Santiago, Chile; ^5^Laboratory of Human Performance, Quality of Life and Wellness Research Group, Department of Physical Activity Sciences, Universidad de Los Lagos, Osorno, Chile; ^6^Institute of Sport Sciences, University of Lausanne, Lausanne, Switzerland; ^7^Laboratory of Cardiorespiratory Control, Department of Physiology, Pontificia Universidad Católica de Chile, Santiago, Chile; ^8^Centro de Envejecimiento y Regeneración (CARE), Pontificia Universidad Católica de Chile, Santiago, Chile; ^9^Centro de Excelencia en Biomedicina de Magallanes (CEBIMA), Universidad de Magallanes, Punta Arenas, Chile; ^10^Centro de Fisiología y Medicina de Altura, Facultad de Ciencias de la Salud, Universidad de Antofagasta, Antofagasta, Chile

**Keywords:** apnea, autonomic nervous system, chemosensitivity, hypoxia, swimming

## Abstract

During an apnea, changes in PaO_2_ activate peripheral chemoreceptors to increase respiratory drive. Athletes with continuous apnea, such as breath-hold divers, have shown a decrease in hypoxic ventilatory response (HVR), which could explain the long apnea times; however, this has not been studied in swimmers. We hypothesize that the long periods of voluntary apnea in swimmers is related to a decreased HVR. Therefore, we sought to determine the HVR and cardiovascular adjustments during a maximum voluntary apnea in young-trained swimmers. In fifteen trained swimmers and twenty-seven controls we studied minute ventilation (V_*E*_), arterial saturation (SpO_2_), heart rate (HR), and autonomic response [through heart rate variability (HRV) analysis], during acute chemoreflex activation (five inhalations of pure N_2_) and maximum voluntary apnea test. In apnea tests, the maximum voluntary apnea time and the end-apnea HR were higher in swimmers than in controls (*p* < 0.05), as well as a higher low frequency component of HRV (*p* < 0.05), than controls. Swimmers showed lower HVR than controls (*p* < 0.01) without differences in cardiac hypoxic response (CHR). We conclude that swimmers had a reduced HVR response and greater maximal voluntary apnea duration, probably due to decreased HVR.

## Introduction

Water sports are part of Olympic Games and have several modalities, including sailing, apnea dives, swimming, among others (FINA, 2020). Swimmers and apnea divers are characterized by the ability to modulate their breathing to such a point that ventilation can be interrupted by ∼4 min (Heusser et al., 2009). Despite that the end of an apnea is related to the central command of the ventilation (Guyenet and Bayliss, 2015), most of the evidence related to regulatory responses to apnea are focused on cardiovascular responses, which has been linked to the sports performance (Costill et al., 1991; Rodríguez-Zamora et al., 2012; Guimard et al., 2014; Costa et al., 2015; Viana et al., 2019). However, considering that PaO_2_ decreases and PaCO_2_ increases during apneas (Muth et al., 2003) and, that respiratory regions, at the brainstem level, could modulate the cardiovascular autonomic responses (Andrade et al., 2018; Díaz et al., 2020), the longer duration of apnea and cardiovascular adjustments to the apnea observed in swimmers, may be related to neural control of breathing, through a desensitization of peripheral chemoreceptors to hypoxia and hypercapnia. However, it has not been studied yet in swimmers.

Most water sports involve immersion periods with both, intermittent (i.e., swimming) and prolonged apneas (i.e., breath-hold dive or speed swimming), and these can last from seconds to minutes depending on discipline (Craig et al., 1985). In fact, one of the most important physiological adaptations, that help improve the athletes’ performances, is the increase of apnea time (Shei, 2018). During a breath-hold there is a decrease in PaO_2_ and increase in PaCO_2_, which could stimulate peripheral and central chemoreceptors (Dempsey et al., 2012; Kumar and Prabhakar, 2012; Guyenet and Bayliss, 2015), suggesting that the end of voluntary apnea is related to the activation of these reflex arcs. The principal peripheral chemoreceptors are allocated in the carotid body (CB), which propagates information to the brainstem in response to several stimuli (PaO_2_, PaCO_2_, pH, flow, glucose, among others) (Iturriaga and Alcayaga, 2004; Kumar and Prabhakar, 2012; Del Rio et al., 2017). The increase in CB chemoreceptors activity elicits an increase in minute ventilation (V_*E*_), sympathetic activity, arterial blood pressure (BP), and heart rate (HR) to cope with metabolic demands (Iturriaga et al., 2016). Therefore, as swimmers are subjected to intermittent apneas during training session, it is possible that the apnea-dependent repetitive hypoxic-hypercapnic stimuli could induce a depressed respiratory neuroplasticity (Vermeulen et al., 2020) and therefore impact on peripheral chemoreflex drive and possibly on apnea time.

Thus, considering that elite apnea divers displayed longer apnea duration (Heusser et al., 2009), it is possible that in swimmers, during a breath-hold the peripheral chemoreceptors could be desensitized to hypoxia and hypercapnia, delaying the end of the apnea, contributing to a prolonged maximum voluntary apnea duration (Lemaître et al., 2009). Thus, we sought to determine the maximum voluntary apnea duration and hypoxic ventilatory response (HVR), on top of the cardiovascular and autonomic adjustments during the breath-hold and during the hypoxic challenge in young-trained swimmers compared to control participants. We hypothesized that sub-aquatic training induces an adaptive phenomenon of peripheral chemoreflex desensitization, decreasing its response in apnea-induced hypoxia/hypercapnia allowing swimmers to have longer apnea times.

## Materials and Methods

### Ethics Statements

Protocols were approved by the Ethical Committee of the Universidad Mayor (Approval number #169_2019) and were performed according to the standards set by the latest version of the Declaration of Helsinki. Participants were carefully informed about the experimental procedures, and the possible risks and benefits associated with their participation in the study. Thereafter, written informed assent and consent were obtained from the parents of under-age athletes and from adult athletes, respectively.

### Subjects

Fifteen young trained regional- to national-level competitive swimmers [eight males and seven females; age, 20.93 ± 5.18 years; height, 169.53 ± 10.44 cm; body mass, 71.5 ± 12.77 kg; body mass index (BMI), 24.7 ± 2.05 kg/m^2^], with 5–12 years of swimming training, and a mean weekly training volume of ∼4 h per day, five times per week, participated in this study. Twenty-seven controls (22 males and five females; age, 17.22 ± 2.42 years; height, 169.52 ± 8.12 cm; body mass, 63.85 ± 10.3 kg; BMI, 22.12 ± 2.49 kg/m^2^) also volunteered to participate in this study. All females were assessed (by a female technician) during the early follicular phase of their menstrual cycle. Experiments were conducted between 08:00 and 17:00 h. Forty-eight hours before experiments participants, were asked to avoid consumption of alcohol, cigarettes, caffeine, or drugs that may alter autonomic control. None of the participants were taking any medication or had a personal or family history of any cardiac, ventilatory or endocrine disorder.

### Experimental Design

A descriptive cross-sectional study was performed to determine HVR and autonomic response to hypoxia and maximum voluntary apnea in young highly trained swimmers compared to controls. The inclusion criteria were: (i) high-performance swimmers with less than 12 years of training; (ii) from national or university teams, active participants in national or international competitions; and a minimum of 20 h of training per week. Exclusion criteria were: (i) potential medical problems or history of cardiorespiratory diseases; (ii) any cardiovascular or respiratory surgery in the past 2 years; (iii) autonomic control impairment at rest, estimated by heart rate variability (HRV) disturbances (low to high frequency ratio of HRV < 2.3) (Nunan et al., 2010); (iv) being in the course of an acute illness or consumption of any drug or pharmacological ergogenic aid and (v) history of chronic obstructive or restrictive pulmonary diseases and/or altered spirometry on the day of the testing session, including (vi) forced expiratory volume at first second (FEV1)/vital capacity (VC) < 70, (vii) FEV1 < 80% of predicted value, or (viii) VC < 80% of predicted value (see Supplementary Table 1).

**FIGURE 1:**
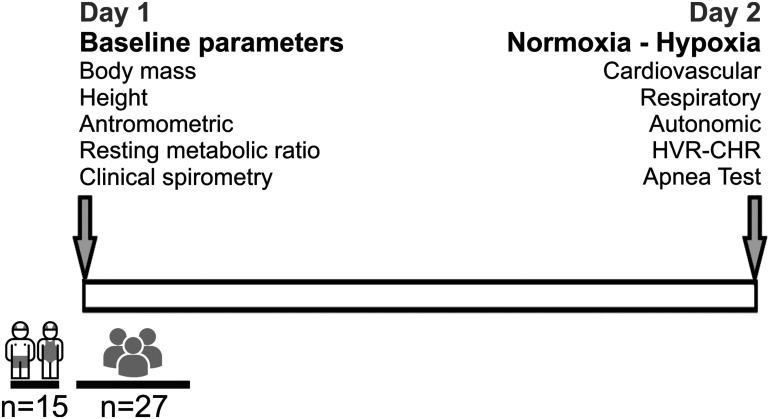
Timeline and design of the experiment. Fifteen young swimmers and twenty-seven control subjects were enrolled to participate in this study. Measurements were performed in 2 days. At day 1, baseline parameters (height, weight, anthropometric, resting metabolic rate, and clinical spirometry were performed. At day 2, respiratory, autonomic, cardiovascular parameters, hypoxic ventilatory response (HVR) and cardiac hypoxic response (CHR), and a maximum apnea duration test were estimated.

On the first day, body mass, height, HR, clinical spirometry [VC; peak expiratory flow (PEF); FEV1; FEV1/VC; forced expiratory flow at 25% (FEF 25), 50% (FEF 50), and 75% (FEF 75) of VC] and resting metabolic ratio were measured (Figure 1). Body mass was estimated to the nearest 0.1 kg using a digital scale (BF-350, Tanita, IL, United States). Height was measured using a wall-mounted stadiometer (HR-200, Tanita, Japan) and recorded to the nearest 0.1 cm. The BMI was calculated as kg/m^2^.

On the second day, the participants were instrumented and positioned in the supine position, at an ambient temperature of ∼22°C. Instrumentation includes: 3-leads electrocardiogram (ECG), core temperature regulator, oxygen saturation using a pulse oximeter measured on the index or thumb finger (SpO_2_) (BK-PO2, BIOBASE, China) and an orofacial mask (Hans Rudolph3, 3700A, Kansas City, MO, United States) connected to a gas mixing chamber to measure airflow and expired gases. From respiratory flow, tidal volume (V_*T*_) was calculated (FE141, ADInstruments Inc., New Zealand). The expired fraction of end-tidal carbon dioxide (F_*E*_CO_2_), end-tidal oxygen (F_*E*_O_2_) levels, and fraction inspired of O_2_ (F_*I*_O_2_) were measured with CO_2_ and O_2_ gas analyzers (ML206, ADInstruments Inc., New Zealand). After instrumenting the subjects, they were given a 15-min rest period in supine position before the recording started. After this period, baseline parameters were recorded for 20 min. In addition, they were instrumented with eye mask and headphones to reduce external noise (MPA 101, Masprot, Chile) to blunt the effect of manipulation of gases on participants’ arousal. Ventilatory data, pulse oximeter, and gas exchanges were digitized using PowerLab Data Acquisition System (PowerLab, 16SP, ADInstruments Inc., New Zealand) and analyzed with LabChart 8.0 (ADInstruments Inc., New Zealand). All experiments were performed according to the timeline shown in Figure 1.

### Hypoxic Ventilatory Response (HVR) and Maximum Voluntary Apnea Test

Hypoxic ventilatory response was evaluated by a poikilocapnic transient hypoxic challenge similar to the one previously described (Pfoh et al., 2016). Participants underwent three consecutive trials (each trial was separate by 5 min) that consisted of five-breaths of 100% N_2_. N_2_ was blended into a port on the mask through N_2_ tubing. After the application of N_2_, 15 min elapsed until ventilatory parameters returned to baseline levels. Afterward, the subjects changed from a supine to a sitting position, and they were instructed to perform a maximum voluntary apnea (after a maximum inspiration). This experiment was performed with a PowerLab Data Acquisition System (PowerLab, 16SP, ADInstruments Inc., New Zealand).

### Cardiac Hypoxic Response (CHR)

Cardiac hypoxic response was assessed by the same stimulus of HVR, similar as in previous experiments (Richalet et al., 2012; Bourdillon et al., 2014). Briefly, three transient tests of five-breaths at 100% N_2_ were applied with 5 min of rest between trials. The highest HR response from these trials was used. To determine the CHR, 1 min of rest in normoxia and 1 min of maximum HR response in hypoxia were used for the analysis. Thus, CHR was calculated as follows: resting HR – maximum HR/SpO_2_ in normoxia – SpO_2_ in hypoxia (= ΔHR/ΔSpO_2_) (Richalet et al., 2012; Bourdillon et al., 2014).

### Electrocardiogram (ECG)

The 3-leads ECG was recorded using a bio-amplifier connected to a digital recording system (PowerLab, 16SP, ADInstruments Inc., New Zealand). The electrodes (3M, Saint Paul, MN, United States) were placed in second derivative (DII) from Einthoven triangle with participants in a supine position. The ECG was recorded continuously, along with breathing gases and ventilation in all experiments considering peripheral chemoreflex test and maximum voluntary apnea test. The sampling frequency was set at 4 kHz and was amplified x100. The ECG analysis was performed with LabChart software v.8 (ADInstruments Inc., New Zealand).

### Heart Rate Variability (HRV)

The HRV was assessed as an indirect measure of autonomic balance of the heart (Camm, 1996). From the 3-lead ECG recording, time series were obtained from R-R interval and a time-varying spectrogram was used to obtain the power spectral density (PSD) of HRV (2 s resolution, Tarvainen et al., 2014). Cut-off frequencies were defined as very low frequency [Very low frequency (VLF_*HRV*_; 0.00–0.04 Hz), low frequency (LF_*HRV*_; 0.04–0.10 Hz), and high frequency (HF_*HRV*_; 0.10–0.40 Hz) (Yuda et al., 2018)]. Additionally, we used the LF/HF_*HRV*_ ratio as an indicator of global autonomic balance of the heart. The LF_*HRV*_ and HF_*HRV*_ were expressed as normalized units (n.u.), calculated as follow: LF n.u. = LF power/(total power – VLF); and HF n.u. = HF power/(total power – VLF) (Camm, 1996); thereafter the area under curve (AUC) of the total responses was calculated, as previously described (Andrade et al., 2020). The baseline values and the maximum apnea event were analyzed using Kubios HRV Premium Software v3.1 (Kubios, Kuopio, Finland).

### Resting Metabolic Rate

Resting metabolic rate (RMR) was measured by indirect calorimetry as previously described (Speakman and Selman, 2003). All participants were instructed to minimize movement after waking-up and to avoid vigorous exercise before the implementation of the calorimetry (Speakman and Selman, 2003; Compher et al., 2006; Nieman et al., 2006). Participants underwent RMR evaluation between 8:00 and 10:00 am. During RMR measurement participants breathed through an oronasal mask (7450 Series Silicone V2, Hans Rudolph, Kansas City, MO, United States) for expired gas collection and analysis (Quark CPET metabolic cart; COSMED, Rome, Italy). Every three measurements the metabolic cart was re-calibrated with a known calibration gas (O_2_ 15%, CO_2_ 5%, N_2_ balanced) (Nieman et al., 2013). The RMR measurement was performed in a specially conditioned room isolated from noise, at a temperature of 23°C and 50% of humidity. Before the measurement, the participants rested for 30 min. The subjects were instrumented and placed in a supine position during the 40 min of measurements. From the total recording, the first 5-min were discarded as part of the acclimatization period. The calculation of respiratory quotient (RQ), protein, carbohydrates and lipids oxidation were obtained from the remaining 35 min. Protein, carbohydrates and lipids oxidation, were expressed as kcal/day and as % of total resting metabolic rate. The recording and analysis were performed with OMNIA, Cardiopulmonary Diagnostic Suite v 1.4 (Quark CPET metabolic cart; COSMED, Rome, Italy).

### Pulmonary Function

Pulmonary function was assessed according to the American Thoracic Society and the European Respiratory Society Task Force consensus (Miller et al., 2005). Briefly, all participants were asked to exert maximum effort during forced breathing. For V_*T*_ subjects were asked to perform a maximal inspiration (inspiratory reserve volume), return to tidal volume, and then perform a maximal expiration (expiratory reserve volume). We used the maximal expiratory curve to calculate VC, PEF, PIF, FEV1, FEV1/VC, FEF 25, FEF 50, and FEF 75. Each subject was asked to do three attempts, from which the best maximal expiratory curve was used for analysis (Andrade et al., 2020). The recording and analysis were performed with OMNIA, Cardiopulmonary Diagnostic Suite v 1.4 (Quark CPET metabolic cart; COSMED, Rome, Italy).

### Statistical Analysis

Data was expressed as mean ± standard deviation (SD). Normality of the data was assessed using Shapiro Wilk test and the homoscedasticity of the variance was determined by Levene’s test. Differences between groups were assessed using unpaired *T*-tests for variables with normal distribution and Mann–Whitney test for variables with non-normal distribution. For the analysis between normoxia and hypoxia the data between both groups was analyzed using a two-way ANOVA followed by the Holm–Sidak *post hoc* test (GraphPad Prism software Inc., version 8.0, La Jolla, CA, United States). Significant differences were set at *p* < 0.05.

## Results

### Baseline Cardiorespiratory and Pulmonary Parameters

At baseline, demographic, respiratory, cardiovascular, and metabolic variables were not different between swimmers and controls, with the exception of inspiratory time (T_*i*_) (Table 1 and Supplementary Table 1). Indeed, swimmers showed greater T_*i*_ compared to controls (1.97 ± 0.89 vs. 1.73 ± 0.25 s, *p* < 0.05) (Table 1). Regarding pulmonary function, swimmers and control participants did not show significant differences (Supplementary Table 1).

**TABLE 1:** Basal anthropometric, respiratory, and cardiovascular characteristics of swimmers compared to control participants at rest condition.

	**Swimmers (*n* = 15)**	**Control (*n* = 27)**
Age (years)	20.93 ± 5.18	17.22 ± 2.42
Weight (kg)	71.50 ± 12.77	63.85 ± 10.31
Height (cm)	169.53 ± 10.44	169.52 ± 8.12
BMI (kg/m^2^)	24.70 ± 2.05	22.12 ± 2.49
**Respiratory**		
VO_2_ (ml/min)	313.68 ± 85.44	326.59 ± 54.90
VCO_2_ (ml/min)	258.49 ± 61.41	275.66 ± 49.48
RQ	0.84 ± 0.10	0.85 ± 0.06
O_2_exp (ml)	102.38 ± 24.88	98.66 ± 21.62
CO_2_exp (ml)	24.39 ± 5.85	25.19 ± 6.43
PetO_2_ (mmHg)	99.46 ± 4.94	99.26 ± 3.53
PetCO_2_ (mmHg)	34.34 ± 3.31	35.56 ± 2.28
F_*i*_O_2_ (%)	20.62 ± 0.16	20.63 ± 0.15
F_*i*_CO_2_ (%)	0.20 ± 0.07	0.25 ± 0.12
SpO_2_ (%)	98.00 ± 0.00	98.15 ± 0.91
T_*i*_ (s)	1.97 ± 0.89*	1.73 ± 0.25
T_*e*_ (s)	2.47 ± 0.95	2.28 ± 0.39
T_*tot*_ (s)	4.44 ± 1.82	4.01 ± 0.57
**Cardiovascular**		
HR (betas/min)	66.51 ± 9.50	60.38 ± 8.15
R-R (s)	0.87 ± 0.12	0.94 ± 0.12

Values are means ± SD. BMI, body mass index; V_*E*_, minute ventilation; RF, Respiratory frequency; V_*t*_, Tidal volume; VO_2_, oxygen uptake; VCO_2_, carbon dioxide production; RQ, respiratory quotient; PetO_2_, end-tidal oxygen pressure; PetCO_2_, end-tidal CO_2_ pressure; F_*i*_O_2_, concentration of inspired oxygen; F_*i*_CO_2_, concentration of inspired carbon dioxide; SpO_2_, oxygen saturation; T_*i*_, Inspiratory time; T_*e*_, expiratory time; T_*tot*_, total time of one breath; HR, Heart rate; R-R, time between ECG peak R; Data with non-normal distribution was analyzed using non-parametric Mann–Whitney test. Data with normal distribution was analyzed using parametric unpaired *T*-test. *P < 0.05 vs. Control group.

### Maximum Voluntary Apnea Time and Cardiovascular Response to Hypoxia and Apnea

**FIGURE 2:**
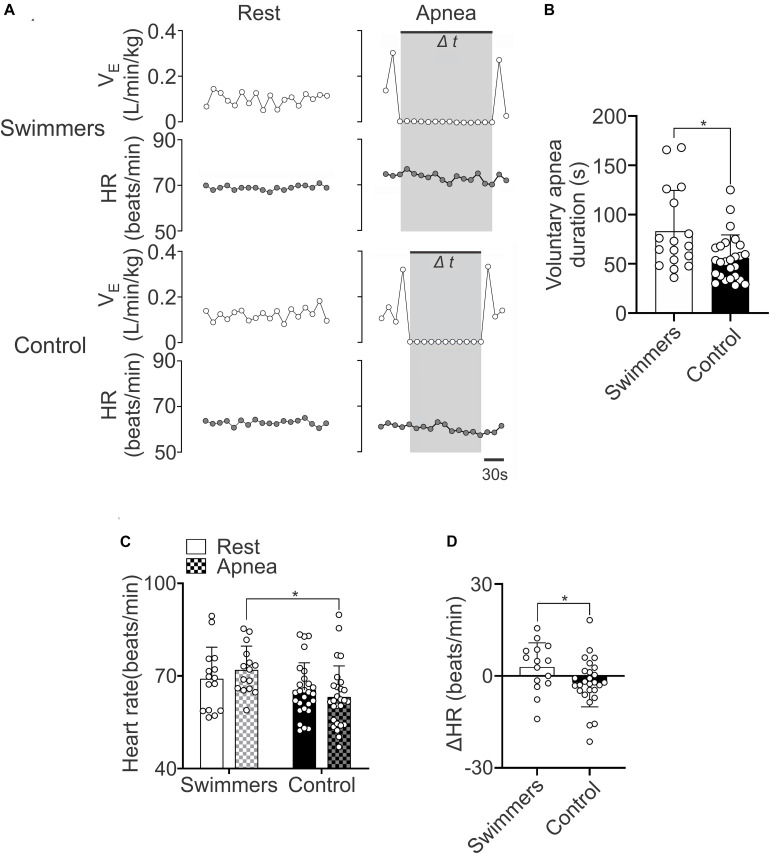
Swimmers are able to maintain longer apnea time and a higher heart rate response during the apnea effort. **(A)** Representative data of V_*E*_ and HR of one swimmer and one control participant during rest and maximal voluntary apnea effort. Note that swimmers displayed higher HR responses than controls during apnea test. **(B)** Summary data of maximal apnea duration. Note that swimmer athletes displayed a marked longer apnea duration compared to control participants. **(C,D)** Summary results of HR and ΔHR during rest and apnea effort. Note that swimmer athletes displayed a more pronounced positive chronotropic response to a maximum voluntary apnea effort. Two-way ANOVA with repeated measures followed by Holm–Sidak *post hoc* test for **(C)** and unpaired *T*-test for **(B,D)**. Values are mean ± SD, Swimmers *n* = 15, Control *N* = 27. *p < 0.05 compared to Swimmers.

The maximum voluntary apnea duration was higher in swimmers than in control participants (83.18 ± 41.43 vs. 55.77 ± 23.71 s) (Figures 2A,B). The HR response during the apnea was higher in swimmers compared to the controls (HR: 71.99 ± 7.67 vs. 63.20 ± 10.07 beats/min) (Figure 2C). In addition, the maximum HR response to voluntary apnea was higher in swimmers compared to controls (ΔHR: 2.94 ± 7.88 vs. −2.20 ± 7.86 Δbeats/min) (Figure 2D). Which showed that the swimmers displayed the classical adaptation to their training (Shei, 2018).

### Autonomic Control During Maximum Voluntary Apnea Test

**FIGURE 3:**
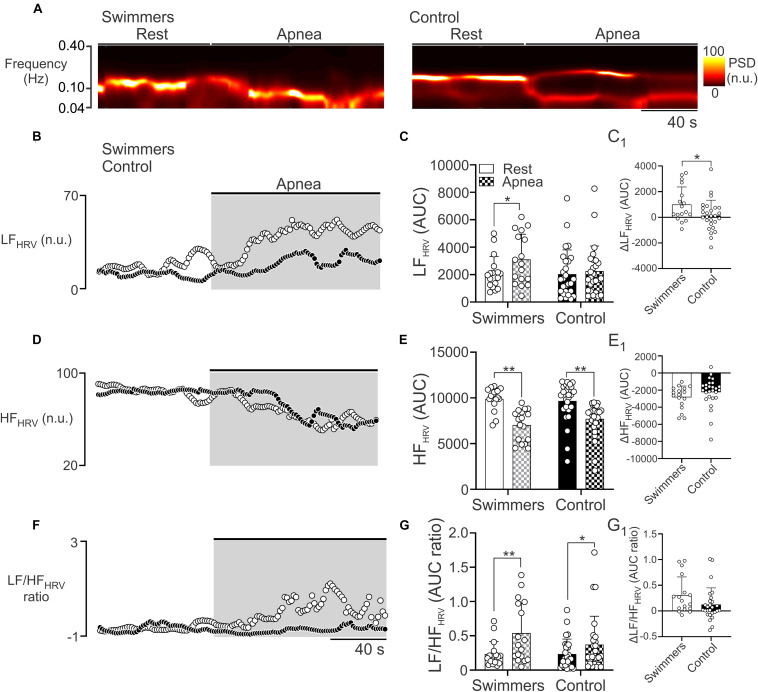
Autonomic control during the maximum voluntary apnea test in swimmers and control participants. **(A)** Representative time-varying domain spectrum of heart rate variability (HRV) during rest and during the maximal voluntary apnea. Note that swimmers athletes displayed a large increase in the low frequency component of HRV (LF_*HRV*_, 0.04–0.10 Hz), concomitant to a decrease of high frequency component of HRV (HF_*HRV*_, 0.10–0.40 Hz) following the apnea effort. **(B,D,F)** Summary data of power spectral density (PSD) of non-stationary analysis of HR with 2-s resolution (time varying domain of HR variability) during the apnea test to LF_*HRV*_, HF_*HRV*_, and LF/HF_*HRV*_ ratio, respectively. **(C,E,G)** Summary data of LF_*HRV*_, HF_*HRV*_, and LF/HF_*HRV*_ ratio, respectively, of HRV non-stationary analysis. **(C_1_,E_1_,G_1_)** Summary data of ΔLF_*HRV*_, ΔHF_*HRV*_, and ΔLF/HF_*HRV*_ ratio, respectively. Two-way ANOVA with repeated measures followed by Holm–Sidak *post hoc* test for **(C,E,G)**; unpaired *T*-test for **(C_1_,E_1_,G_1_)**. Values are mean ± SD, Swimmers *n* = 15, Control *N* = 27. *p < 0.05; **p < 0.01.

During the maximum voluntary apnea test, the LF_*HRV*_ in swimmers was increased compared to the resting condition (Figures 3A–C). However, the control participants did not show significant changes in LF_*HRV*_, between rest and the apnea test (*p* = 0.424) (Figure 3C). The ΔLF_*HRV*_ was higher in swimmers than in controls (999.2 ± 1368 vs. 140.4 ± 1194 ΔAUC, *p* = 0.033) (Figure 3C_1_). Regarding to HF_*HRV*_ during apnea, both groups showed a decrease of parasympathetic drive (*p* < 0.001), without differences with their respective resting values (*p* = 0.224) (Figures 3A,D,E,E_1_). Both groups showed an increase in LF/HF from rest to apnea, (swimmers: 0.23 ± 0.18 to 0.54 ± 0.44, *p* < 0.001; controls: 0.23 ± 0.22 to 0.37 ± 0.41, *p* = 0.037). The magnitude of the LF/HF increase was not larger in swimmers than in controls (0.31 ± 0.35 vs. 0.12 ± 0.32; *p* = 0.087) (Figures 3F,G,G_1_).

### Ventilatory and Cardiac Response to Hypoxia

**FIGURE 4:**
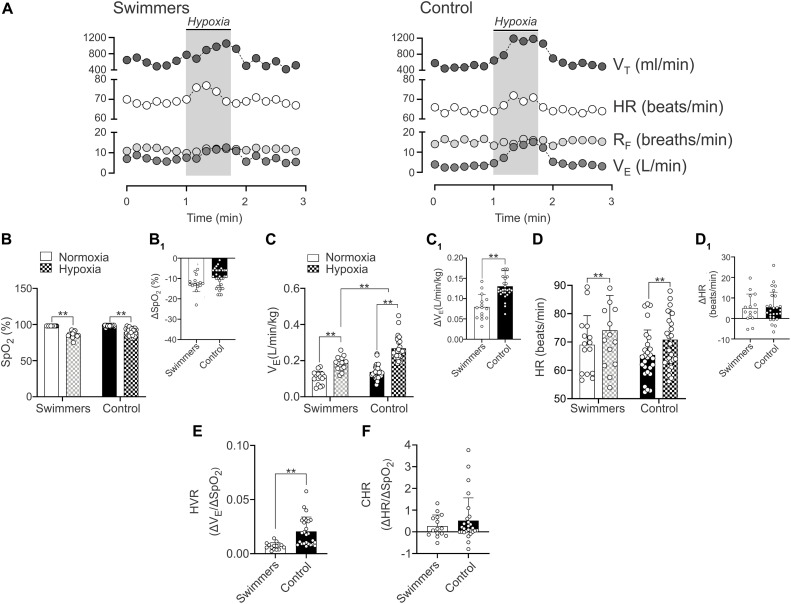
Hypoxic ventilatory (HVR) cardiac (HCR) responses in young trained swimmers. **(A,B)** Representative data of V_*T*,_ HR, R_*F*_, and V_*E*_ of one Swimmer and Control participant during a hypoxic challenge (pure N_2_). Note that swimmers displayed a decrease V_*E*_ response compared to the control participants. **(B,B_1_)** Summary of SpO_2_ changes in normoxia and hypoxia and summary of ΔSpO_2_ between normoxia and hypoxia in both groups, respectively. **(C)** Summary of V_*E*_ responses to hypoxia of both groups. Note that the breathing response was minor in swimmers compared to the control participants. **(C_1_)** Quantification of ΔV_*E*_ between normoxia and hypoxia in both groups. **(D)** Summary of HR responses to hypoxia of both groups. **(D_1_)** Quantification of ΔHR between normoxia and hypoxia in both groups. **(E)** Swimmers group showed a significantly lower HVR compared to controls. **(F)** Summary data of CHR. Two-way ANOVA with repeated measures followed by Holm–Sidak *post hoc* test for **(B–D)**; unpaired *T*-test for **(C_1_)**; and Mann–Whitney test for **(D_1_,E,F)**. Values are mean ± SD, Swimmers *n* = 15, Control *N* = 27. **p < 0.01.

The HVR and HCR responses are shown in Figure 4 and Table 2. In normoxia there were no differences between swimmers and controls in SpO2 (98.0 ± 0.0 vs. 98.1 ± 0.9%) (Figures 4B,B_1_ and Table 2) and V_*E*_ (0.11 ± 0.04 vs. 0.14 ± 0.04 L/min/kg) (Figures 4C,C_1_). In addition, before chemoreflex test (during normoxia) R_*F*_ and V_*T*_ weren’t different between groups (Table 2).

**TABLE 2:** Cardiovascular and respiratory responses to severe hypoxic challenge in swimmers and control participants.

	**Swimmers (*n* = 15)**		**Controls (*n* = 27)**		**Swimmers**	**Controls**
	**Normoxia**	**Hypoxia**	**Normoxia**	**Hypoxia**	**Δ Normoxia-hypoxia**	**Δ Normoxia-hypoxia**
R_*F*_ (breaths/min)	12.90 ± 3.91	12.67 ± 3.74	15.45 ± 2.81	15.86 ± 3.59	−0.23 ± 2.3	0.42. ± 1.94
V_*T*_ (ml)	627.26 ± 321.67	1124.85 ± 416.94*	583.55 ± 194.72	1124.74 ± 372.58*	497.59 ± 270.41	541.18 ± 238.63
V_*T*_ (ml/kg)	8.68 ± 3.74	15.82 ± 5.61*	9.18 ± 2.87	17.75 ± 5.78*	7.14 ± 4.02 +	8.57 ± 3.84
VO_2_ (ml/min)	317.79 ± 147.02	1097.12 ± 354.01*	328.10 ± 96.48	1129.58 ± 257.25*	779.33 ± 227.3	712.43 ± 319.76
VO_2_ (ml/kg/min)	4.48 ± 1.63	15.69 ± 4.14*	5.16 ± 1.62	17.91 ± 5.35*	11.20 ± 2.95	11.32 ± `5.75
VCO_2_ (ml/min)	326.04 ± 125.51	407.12 ± 101.32*	306.84 ± 84.3	418.24 ± 112.61*	81.08 ± 95.13 +	99.08 ± 81.3
VCO_2_ (ml/kg/min)	4.65 ± 1.46	5.84 ± 1.16*	4.78 ± 1.19	6.52 ± 1.63*	1.19 ± 1.4	1.55 ± 1.18
F_*E*_O_2_	16.55 ± 0.77	12.91 ± 1.65*	16.75 ± 0.53	13.72 ± 1.76*	−3.64 ± 1.27	−3.03 ± 1.54
F_*E*_CO_2_	4.53 ± 0.54	4.03 ± 0.49	3.88 ± 0.8	3.45 ± 0.81	−0.49 ± 0.23	−0.43 ± 0.26

Values are means ± SD. R_*F*_, Respiratory frequency; V_*T*_, Tidal volume; VO_2_, oxygen consumption; VCO_2_, carbon dioxide production; F_*E*_O_2_, fractional content of oxygen; F_*E*_CO_2_, fractional content of carbon dioxide. Unpaired *T*-Test and *U*-Mann Whitney Test were used to parametric and non-parametric variables, respectively. Two-way ANOVA with repeated measures followed by Holm–Sidak *post hoc* test was performed. *P < 0.05 vs. Normoxia; +*P* < 0.05 vs. Controls.

#### Hypoxic Ventilatory Response

Both swimmers and controls showed a significant decrease of SpO_2_ (*p* < 0.001), without differences between groups (Figure 4B). Indeed, the ΔSpO_2_ was not different between groups (*p* = 0.089) (Figure 4B_1_). Swimmers showed a lower increase in V_*E*_ (0.11 ± 0.04 vs. 0.19 ± 0.04 L⋅min⋅kg^–1^) from normoxic to hypoxic condition than the controls (0.14 ± 0.04 vs. 0.27 ± 0.06 vs. L/min/kg) (Figure 4C). Consequently, the ΔV_*E*_ was lower in swimmers compared to control participants (5.51 ± 0.49 vs. 8.14 ± 0.34 Δ L⋅min⋅kg^–1^) (Figure 4C_1_) and the HVR, expressed as ΔV_*E*_/ΔSpO_2_ was significantly lower in swimmers compared to the control participants (0.007 ± 0.001 vs. 0.016 ± 0.002 ΔV_*E*_/ΔSpO_2_) (Figure 4E).

During the chemoreflex test (hypoxic challenge), swimmers and controls participants did not show significant differences in R_*F*_ and F_*E*_CO_2_, while there were significant increases in absolute and relative V_*T*_, absolute and relative VO_2_, absolute and relative VCO_2_ and F_*E*_O_2_ in both groups. However, the changes were only different for relative V_*T*_ and relative VCO_2_ (Table 2).

#### Hypoxic Cardiac Response

Both groups showed a significant increase of maximum HR during hypoxic stimulation compared to their normoxic condition (swimmers: 74.12 ± 12.30 vs. 69.06 ± 10.26 beats/min; controls: 70.81 ± 9.97 vs. 65.40 ± 8.86 beats/min, *p* < 0.001) (Figure 4D). Indeed, cardiac response to hypoxia was similar in swimmers (5.1 ± 6.9 Δbeats/min) compared to controls (5.4 ± 7.4 Δbeats/min) (Figure 4D_1_). Accordingly, the HCR was similar between swimmers (0.27 ± 0.51 ΔHR/ΔSpO_2_) and controls (0.52 ± 1.04 ΔHR/ΔSpO_2_) (Figure 4F). In addition, during hypoxia there were no significant differences between groups in HRV disturbances (Supplementary Table 2).

## Discussion

The aim of the study was to determine the maximum voluntary apnea duration and HVR, and the cardiovascular and autonomic adjustments during a breath-hold and during the hypoxic challenge in young-trained swimmers compared to control participants. The main findings of the present study are as follows: when compared to controls, swimmers showed: (i) longer maximum voluntary apnea duration; (ii) markedly decrease of HVR, with similar CHR; (iii) higher cardiac response during the maximum voluntary apnea test characterized by an overall autonomic imbalance. Our results strongly suggest that the lower ventilatory response to hypoxia (determined through a hypoxic challenge) might contribute to longer apnea duration in swimmers. However, the cardiovascular and autonomic adjustments during the breath-hold could be independent of hypoxic chemoreceptor activity and would be attributed to other mechanisms at the central level (i.e., central chemoreceptors).

### Hypoxic Ventilatory Response and Apnea Duration in Young Highly Trained Swimmers

Aquatic immersion sports, such as apnea diving, artistic or distance swimming involve severe cardiovascular and autonomic responses to maintain blood flow and oxygen supply to activated tissues during exercise, despite not having pulmonary ventilation for several seconds or minutes (Heusser et al., 2009). During apneas, the decrease and increase of PaO_2_ and PaCO_2_ (Muth et al., 2003; Mansukhani et al., 2015), respectively, could stimulate peripheral chemoreceptors (Dempsey et al., 2012; Kumar and Prabhakar, 2012; Guyenet and Bayliss, 2015), and contribute to the end of apnea. Accordingly, considering that swimmers athletes are adapted to extreme hypoxia and hypercapnia during exercise (Heusser et al., 2009; Breskovic et al., 2010), we proposed that HVR could be diminished in young-trained swimmers. Thus, our data showed that swimmers displayed a decrease of HVR response, stimulated by a hypoxic challenge, which could contribute to maintain the apnea duration. We found that swimmers displayed a longer voluntary apnea duration compared to the control participants as shown previously (Heusser et al., 2009). This phenomenon is a typical adaptation in swimmers’ athletes (Schagatay et al., 2000). However, it is important to mention that we observed ∼80-s of maximum apnea duration, a “low” value compared to apnea divers (Heusser et al., 2009). Apnea divers are able to maintain a voluntary apnea up to ∼240-s (Heusser et al., 2009). This marked difference could be related to training status, specific training adaptations or genetics, such as the size of the spleen seen in immersion hunter tribes (Ilardo et al., 2018) or as well as the experience related to maintain longer apnea efforts (Viana et al., 2019). Nevertheless, and contrarily to our data, it has been shown that the breathing responses to hypoxia were not different between apnea divers and control subjects (Dujic et al., 2008; Breskovic et al., 2010). This may be explained by the training adaptations that are specific to swimming. Similarly, Bruce et al. (2021) showed that the maximal voluntary apnea duration is dependent of inspired air prior to apnea, but the HVR was not associated to chemoreflex drive. This finding is contrarily to our data; however, it is important to mention that in the present study we used young-trained swimmers and not healthy individual, which does not reflect the physiological adaptations to underwater training.

In addition, we observed that HCR was similar between swimmers and controls, suggesting that the chronotropic response to hypoxic stimulation is independent to HVR in swimmers. Nevertheless, similar to our results, Dujic et al. (2008) found that repeated episodes of hypoxemia alone are not sufficient to drive an increase in resting sympathetic activity in breath-hold divers. In addition, Breskovic et al. (2010) showed no differences in muscle sympathetic nerve activity during hypoxic challenge between divers and control participants, which could explain partly the HR response. Indeed, contrarily to apnea divers who sustain prolonged apneas, swimmers performed several intermittent apnea efforts during training sessions [between 3 and 20 s depending on the competition (Guimard et al., 2014)] which could be associated to a reduced HVR, but with similar HCR.

During an apnea, the PaCO_2_ increases and pH decreases can be sensed by the central chemoreceptors located in the brainstem. The most important zone in the brainstem, related to autonomic and breathing responses to CO_2_, is the retrotrapezoid nucleus (RTN) (Guyenet and Bayliss, 2015; Díaz et al., 2020). Therefore, it is possible that in swimmers the retention of CO_2_ during the apnea, triggered an RTN-dependent sympathoexcitation, which may have induced a greater tachycardic response. However, controversial evidence has been observed. Indeed, Trembach and Zabolotskikh (2017) showed that peripheral chemosensitivity, through stimulation by transient CO2 inhalation, was associated to the maximum voluntary apnea in healthy individuals; however, Bain et al. (2017) showed that the central respiratory chemoreflex, through stimulation by hyperoxic rebreathe, was not related to maximum breath-hold duration. The divergent evidence depicted by the manuscript of Trembach and Zabolotskikh (2017) compared to Bain et al. (2017) may be partially explained by the characteristics of the participants. While Trembach and Zabolotskikh (2017) recruited healthy participants, Bain et al. (2017) recruited apnea divers. In our study, we included young-trained swimmers’ athletes; hence, it is possible that the sport-training background of the athletes could partially explain the different results reported among studies.

### Autonomic Control During Apnea in Young Highly Trained Swimmers

Acute autonomic response during an apnea effort is critical to maintain the blood flow and oxygen supply to activated muscles during training and/or competitive events in aquatic sports (Heusser et al., 2009; Breskovic et al., 2010). During an apnea, the arterial hypoxemia could explain the positive cardiac inotropic and chronotropic response, mediated by a parasympathetic withdrawal, which could be independent of pulmonary inflation previous to the apnea (inflation reflex) (Siebenmann et al., 2019). However, our data showed that control participants are characterized by a decrease of HF_*HRV*_, mainly associated to the parasympathetic tone, while swimmers showed both, a decrease of HF_*HRV*_ and an increase of LF_*HRV*_, which is influenced by sympathetic and parasympathetic system (Goldstein et al., 2011), during the maximum apnea effort. Furthermore, concomitant with the autonomic response, we observed a more pronounced positive chronotropic response in swimmers than in control participants. Therefore, the more severe tachycardic response in swimmers may be a consequence of the autonomic imbalance due to decreased parasympathetic outflow and an increase of sympathetic drive.

The present study is not free of limitations. All our experiments were performed at rest, which is completely different to an exercise condition. Further, the maximum voluntary apnea efforts were performed outside of the water, which could impact on the perception of the subjects (Rodríguez-Zamora et al., 2012). Therefore, further studies should imply in-water immersion so to adequately reproduce the environment swimmers are exposed to. Furthermore, we did not test the central chemoreflex response, which has been seen to be decreased in swimmers (Ohkuwa et al., 1980) through ventilatory response to CO_2_, which is critical to determine the chronotropic response during the voluntary apnea test, considering that the swimmers showed more retention of CO_2_, suggesting the participation of central chemoreceptors. In addition, our study does not provide a direct relationship between HVR and apnea duration, which could be determined through pharmacological approaches. Therefore, future studies are required to assess the contribution of both, peripheral and central chemoreceptors on cardiac and autonomic responses induced by an apnea in swimmers athletes, who are subjected to different training regimen compared to other water sports.

## Conclusion

Swimmers are characterized by the ability to modulate their breathing and by adaptations to intermittent hypoxemia/hypercapnia. Considering the longer duration of apnea in swimmers and the following cardiovascular adjustments to stop ventilation, we investigated the HVR in trained swimmers. Our data showed that the apnea duration was longer, and that HVR was decreased in swimmers, when compared to control subjects. However, the tachycardic response to apnea was more pronounced in swimmers with more retention of CO_2_, which suggest that central chemoreceptors could be involved in the autonomic response induced by the apnea.

## Data Availability Statement

The raw data supporting the conclusions of this article will be made available by the authors, without undue reservation.

## Ethics Statement

Protocols were approved by the Ethical Committee of the Universidad Mayor (Approval number #169_2019) and were performed according to the standards set by the latest version of the Declaration of Helsinki. Written informed consent to participate in this study was provided by the participants’ legal guardian/next of kin.

## Author Contributions

DA and AA-Á performed the data collection and analysis, performed interpretation of the data, and contributed to the preparation of the manuscript. DA, AA-Á, MV-M, CV, MI, RR-C, CA, GM, RDR, and MI contributed to the preparation of the manuscript. RR-C, MV-M, CA-L, GPM, RDR, and MI performed interpretation of the data and contributed to the preparation of the manuscript. DA contributed to the concept of the project, performed interpretation of the data, preparation of the manuscript, and undertook in the laboratory of data analysis and interpretation. All authors approved the final version of the manuscript.

## Conflict of Interest

The authors declare that the research was conducted in the absence of any commercial or financial relationships that could be construed as a potential conflict of interest.
