# Supplementary material for: Hypoxic Respiratory Chemoreflex Control in Young Trained Swimmers
Source: Front Physiol. 2021 Feb 26;12:632603. doi: 10.3389/fphys.2021.632603 (PMC7953139; doi:10.3389/fphys.2021.632603)
Supplement: Supplementary file 1 [file Table_1.pdf]

Supplemental Material

HYPOXIC RESPIRATORY CHEMOREFLEX CONTROL IN YOUNG TRAINED SWIMMERS

Alexis Arce-Álvarez[1], Carlos Veliz[2], Manuel Vazquez-Muñoz[3,4], Magdalena von Igel[2], Cristian Alvarez-Lepín[5], Rodrigo Ramirez-Campillo[5], Mikel Izquierdo[3], Gregoire P. Millet[6], Rodrigo Del Rio[7,8,9], *David C. Andrade[2,7,10]

[1]Escuela de Kinesiología, Facultad de Salud, Universidad Católica Silva Henríquez, Santiago, Chile. [2]Centro de Investigación en Fisiología del Ejercicio, Facultad de Ciencias, Universidad Mayor, Santiago, Chile. [3]Navarrabiomed, Complejo Hospitalario de Navarra (CHN), Universidad Pública de Navarra (UPNA), IdiSNA, Pamplona, Navarra, Spain. [4]Unidad de Estadística, Departamento de Calidad, Clínica Santa María, Santiago, Chile. [5]Laboratory of Human Performance, Quality of Life and Wellness Research Group, Department of Physical Activity Sciences, Universidad de Los Lagos, Osorno, Chile. [6]Institute of Sport Sciences, University of Lausanne, CH-1015, Lausanne, Switzerland. [7]Laboratory of Cardiorespiratory Control, Department of Physiology, Pontificia Universidad Católica de Chile, Santiago, Chile. [8]Centro de Envejecimiento y Regeneración (CARE), Pontificia Universidad Católica de Chile, Santiago, Chile. [9]Centro de Excelencia en Biomedicina de Magallanes (CEBIMA), Universidad de Magallanes, Punta Arenas, Chile. [10]Centro de Fisiología y Medicina de Altura, Facultad de Ciencias de la Salud, Universidad de Antofagasta, Antofagasta, Chile

Running Head: Respiratory chemoreflex in swimmers

*Corresponding author: David C. Andrade, Ph.D.
Laboratory of Cardiorespiratory Control
Pontificia Universidad Católica de Chile.
Centro de Fisiología y Medicina de Altura
Facultad de Ciencias de la Salud, Universidad de Antofagasta
Antofagasta, Chile.
Tel: +569 88928666
E-mail: dcandrade@uc.cl
david.andrade@uantof.cl

SUPPLEMENTARY TABLES.

Supplementary table 1. Baseline ventilatory function and metabolic parameters in swimmers and control participants.

**Ventilatory**	**Swimmers (n=15)**	**Control (n=27)**
FVC (L)	4.90 ± 1.23	4.96 ± 0.91
PEF (L/s)	7.70 ± 1.92	7.91 ± 1.54
FEV1 (L)	3.98 ± 0.84	4.12 ± 0.7
FEV1/VC (%)	81.93 ± 5.14	83.25 ± 4.8
FEF25 (L/s)	2.01 ± 0.45	2.30 ± 0.66
FEF50 (L/s)	4.65 ± 1.0	4.68 ± 1.23
FEF75 (L/s)	6.78 ± 1.94	6.72 ± 1.44
**Metabolic**		
Fat (%)	62.40 ± 19.59	53.74 ± 18.16
Carbohydrates (%)	41.49 ± 25.39	46.26 ± 18.16
Protein (%)	0.00 ± 0.00	0.00 ± 0.00
Fat (kcal/day)	1419.19 ± 565.46	1218.63 ± 416.48
Carbohydrates (kcal/day)	840.58 ± 408.37	1052.14 ± 436.54
Protein (kcal/day)	0.00 ± 0.00	0.00 ± 0.00

Values are means ± SD. Data with parametric distribution was analyzed using T-test. Data with non-parametric distribution was analyzed using Mann–Whitney test. **FVC**: forced expiratory vital capacity; **PEF**: peak expiratory flow; **FEV1**: forced expiratory volume in one second; **FEF25**, **MEF 25, MEF 50**, and **MEF 75**: mean expiratory flow at 25%, 50%, and 75% of vital capacity, respectively; **VC:** vital capacity. No significant differences were observed between groups.

Supplementary Table 2. Autonomic response to severe hypoxic challenge in swimmer and control participants.

	**Swimmers (n=15)**		**Control (n=27)**		**Swimmers**	**Control**
	Normoxia	Hypoxia	Normoxia	Hypoxia	Δ Normoxia-Hypoxia	Δ Normoxia-Hypoxia
Total Power (ms^2)	6916.25 ± 6118.49	5559.67 ± 5910.83	7423.24 ± 7236.59	5933.25 ± 6753.61	-1356.57 ± 4191.38	-1489.99 ± 8115.13
VLF (ms^2)	1433.52 ± 1123.52	574.02 ± 1026.24 *	1364.78 ± 1480.26	675.98 ± 760.83 *	-859.50 ± 1452.17	-688.79 ± 1492.61
LF (ms^2)	910.36 ± 686.34	631.99 ± 613.88	626.47 ± 573.18	633.76 ± 715.52	-278.37 ± 816.84	7.29 ± 862.69
HF (ms^2)	4373.06 ± 4564.98	4227.5 ± 5069.35	5018.81 ± 6048.47	4130.23 ± 5453.42	-145.81 ± 3000.77	-888.59 ± 7026.54
SDNN (ms)	74.11 ± 36.68	76.39 ± 40.88	110.31 ± 169.95	71.53 ± 34.62	2.29 ± 23.03	-38.78 ± 182.19
RMSSD (ms)	65.74 ± 46.25	64.73 ± 50.0	80.78 ± 49.45	71.63 ± 43.59	-1.01 ± 28.58	-9.15 ± 44.03
PNN50 (%)	32.68 ± 26.78	30.66 ± 29.43	45.58 ± 24.3	42.42 ± 24.12	-2.02 ± 13.91	-3.16 ± 18.35
SD1 (ms)	46.50 ± 32.7	46.18 ± 35.81	57.15 ± 35.0	51.18 ± 31.14	-0.32 ± 20.41	-5.97 ± 31.27
SD2 (ms)	93.05 ± 42.43	96.55 ± 47.99	90.11 ± 43.52	85.58 ± 41.53	3.50 ± 28.24	-4.53 ± 38.95
SD2/SD1	2.45 ± 0.81	1.87 ± 0.79	2.53 ± 0.91	2.01 ± 1.07	-0.07 ± 0.7	0.15 ± 0.75

Values are means ± SD. **VLF**, very low frequency of HRV; **LF**, low frequency component of HRV; **HF**, High frequency component of HRV; **SDNN**, Standard deviation of the NN intervals; **RMSSD**, Root mean square of the successive differences between adjacent normal R-R intervals; **PNN50**, proportion of NN50 divided by the total number of NN intervals; **SD1**, short-term variability of NN intervals; **SD2**, long-term variability of NN intervals. Unpaired T-Test for parametric variables (LF (%), HF (%), PNN50,) and U-Mann Whitney Test for non-parametric variables (Total Power, VLF, LF, LF (n.u.), HF, HF (n.u.), LF/HF, SDNN, RMSSD, SD1, SD2, SD2/SD1) was performed to compare ΔNormoxia-Hypoxia. Two-way ANOVA with repeated measures followed by Holm-Sidak post-hoc test was performed. *, P<0.05 vs Normoxia; + P<0.05 vs Controls.
